# Prevalence and determinants of termination of pregnancy among reproductive-age women who had a short preceding birth interval in Sub-Saharan Africa: a multilevel analysis

**DOI:** 10.3389/fgwh.2024.1471187

**Published:** 2024-11-27

**Authors:** Alebachew Ferede Zegeye, Tadesse Tarik Tamir, Enyew Getaneh Mekonen, Masresha Asmare Techane, Bewuketu Terefe, Belayneh Shetie Workneh

**Affiliations:** ^1^ Department of Medical Nursing, School of Nursing, College of Medicine and Health Sciences, University of Gondar, Gondar, Ethiopia; ^2^ Department of Pediatric and Child Health Nursing, School of Nursing, College of Medicine and Health Sciences, University of Gondar, Gondar, Ethiopia; ^3^ Department of Surgical Nursing, School of Nursing, College of Medicine and Health Sciences, University of Gondar, Gondar, Ethiopia; ^4^ Department of Community Health Nursing, School of Nursing, College of Medicine and Health Sciences, University of Gondar, Gondar, Ethiopia; ^5^ Department of Emergency and Critical Care Nursing, School of Nursing, College of Medicine and Health Sciences, University of Gondar, Gondar, Ethiopia

**Keywords:** determinants, prevalence, pregnancy termination, short birth intervals, Sub-Saharan Africa

## Abstract

**Background:**

Termination of pregnancy is one of the biggest five causes of maternal mortality in countries with low and middle incomes. Although termination of pregnancy is hazardous, its prevalence and determinates are not well studied in developing countries. Therefore, this study aims to assess the prevalence and determinants of termination of pregnancy among reproductive-age women who had a short preceding birth interval in Sub-Saharan Africa.

**Methods:**

Data from the most recent Demographic and Health Surveys, which covered 21 Sub-Saharan African countries from 2015 to 2022, were used for secondary data analysis. The study used a total of 283,785 women. Stata 14 was used to analyze the data. The determinants of termination of pregnancy were determined using a multilevel mixed-effects logistic regression model. Significant factors associated with termination of pregnancy were declared significant at *p*-values < 0.05. The result was interpreted using the confidence interval and adjusted odds ratio. The best-fit model was determined to be the one with the highest log likelihood ratio and the lowest deviance.

**Results:**

In Sub-Saharan Africa, one in ten women with short birth intervals experienced pregnancy termination. Individual factors, including the sex of the preceding birth (AOR = 1.21, 95% CI: 1.05, 1.40), maternal age (AOR = 1.57, 95% CI: 1.27, 1.95), pregnancy complications (AOR = 1.28, 95% CI: 1.09, 1.49), No ANC visits (AOR = 2.29, 95% CI: 1.26, 4.14), previous cesarean section delivery (AOR = 1.74, 95% CI: 1.32, 2.30), <6 months of breastfeeding (AOR = 1.56, 95% CI: 1.35, 1.81), traditional contraception usage (AOR = 1.67, 95% CI: 1.13, 2.46), poor wealth status (AOR = 1.50, 95% CI: 1.22, 1.85), and community-level factors such as urban residence (AOR = 1.31, 95% CI: 1.06, 1.62) had higher odds of pregnancy termination.

**Conclusions:**

This study concludes that termination of pregnancy rates among women with short preceding birth interval is high. The study identified that both individual and community-level variables were determinants of termination of pregnancy. Therefore, the ministries of health in Sub-Saharan African countries should give attention to those women who underutilize antenatal care services and to women from urban areas while designing policies and strategies targeting reducing termination of pregnancy rates.

## Introduction

The World Health Organization (WHO) defines an abortion as the termination of a pregnancy, whether induced or spontaneous, before 20 weeks of gestation (1). Termination of pregnancy is one of the biggest five causes of maternal mortality in countries with low and middle incomes (2). Globally, an estimated 73 million induced abortions have been carried out annually. Six out of 10 (61%) of all unintended pregnancies, and 3 out of 10 (29%) of all pregnancies, end in induced abortion (1, 3).

Short birth intervals are particularly concerning for maternal health as they increase risks of maternal depletion, insufficient recovery time, and heightened susceptibility to adverse outcomes in subsequent pregnancies (4). Short intervals limit time for physiological recuperation, which can lead to complications such as anemia, malnutrition, and heightened risk of uterine rupture, preterm birth, and maternal mortality (5). Evidence suggests that these intervals strain maternal health resources, impacting both physical and mental health recovery. Additionally, closely spaced pregnancies can exacerbate socioeconomic stressors, particularly in resource-limited settings, compounding health risks (6).

Nearly two thirds of termination of pregnancy occurs in developing countries. In Asia, particularly in south and central Asia, unsafe abortions account for more than half of all abortions undertaken (7). The World Health Organization (WHO) defines unsafe abortions as procedures for terminating unintended pregnancies that are carried out either by individuals lacking the necessary skills or in environments that do not conform to minimal medical standards, or both (8, 9). The WHO's recent definition emphasizes that unsafe abortions are a significant but preventable cause of maternal deaths and morbidities, particularly in developing countries where 97% of unsafe abortions occur. Most terminations of pregnancy (about three out of four) are potentially hazardous in Latin America and Africa. The least safe conditions are used for over half of all abortions in Africa (9). An estimated 55.9 million abortions are performed each year, 49.3 million of which take place in developing countries (10).

The prevalence of pregnancy termination practices varies across country, with 3.1% in western Africa and 3.8% in northern Africa (11, 12). Thirty-seven deaths per 100,000 live births in Sub-Saharan Africa account 13% of all maternal deaths worldwide, and are specifically associated with abortions (13). Termination of pregnancy rates remain high in developing countries, especially in Sub-Saharan Africa (SSA), where abortion is legally limited, despite being lower in developed countries where abortion laws have been liberalized and safe abortion services are freely accessible (14, 15). In Sub-Saharan Africa, 77% of abortions are unsafe because they are handled by unskilled individuals and use non-recommended methods. As of 2019, the region experienced about 6.2 million unsafe abortions annually, which have a detrimental effect on people, families, and healthcare systems.

The studies carried out in different parts of the world revealed that termination of pregnancy was significantly associated with women's age (16, 17), residence (18, 19), poor wealth index (20), use of substance (21), educational level (22, 23). Even though the WHO provides global technical and policy guidance on the use of contraception to prevent unintended pregnancy and abortion, maternal deaths related to abortion remain high in developing countries, especially in Sub-Saharan Africa.

Sub-Saharan African countries are actively working to improve family planning services to prevent unintended pregnancies and reduce the need for pregnancy termination. For instance, Angola is investing $500K USD annually to improve access for young people (24). Benin aims to raise its modern contraceptive prevalence rate to 25% by 2022 (25), while Burundi targets a substantial increase to 60% by 2030 (26). Ethiopia has made remarkable progress, reaching a 41% contraceptive rate (27), and Kenya envisions zero unmet needs by 2030 (28).

Despite the above efforts, maternal mortality and morbidity are still highly related to reproductive health. As far as our search of the literature and knowledge is concerned, there has been no study conducted on termination of pregnancy among women who had a short birth interval in Sub-Saharan Africa, a large sample from DHS data. Therefore, this study used multilevel mixed effect analysis of the most recent Demographic and Health Survey to investigate the prevalence and predictors of pregnancy termination among women who had short birth intervals in Sub-Saharan Africa.

## Materials and methods

### Study setting

The Sub-Saharan region of Africa is the part of the continent that is located south of the Sahara and consists of four large and diverse regions, including Eastern Africa, Central Africa, Western Africa, and Southern Africa. The region is tremendously diversified, consisting of low, lower-middle, upper-middle, and wealthy countries. The region constitutes an area of 9.4 million square miles and an estimated number of 407 million by 2030 and then 607 million by 2050 of reproductive-age women (29, 30). This study was conducted based on the recent DHS survey data from twenty one Sub-Saharan African countries such as Angola, Benin, Burundi, Cameron, Ethiopia, Gabon, Gambia, Guinea, Kenya, Liberia, Mali, Malawi, Nigeria, Rwanda, Senegal, Sera lion, Tanzania, Uganda, South Africa, Zambia, and Zimbabwe. Based on the World Bank's income classifications for the fiscal year 2024, those countries are categorized as: low-income countries: Burundi, Ethiopia, Gambia, Guinea, Liberia, Mali, Malawi, Sierra Leone, Tanzania, Uganda, and Zimbabwe (31). Lower-middle-income countries: Angola, Benin, Cameroon, Kenya, Nigeria, Rwanda, Senegal, Zambia (31). Upper-middle-income countries: Gabon, South Africa (31).

### Study design and period

A mixed-effect cross-sectional study with a community-based approach has been carried out. A multilevel mixed effect analysis was undertaken using data from 21 Sub-Saharan African countries for a recent DHS survey that took place between 2015 and 2022. Part of the global Demographic and Health Survey, the Demographic and Health Survey (DHS) is a 5-year national survey project that uses pretested, validated, and structured instruments. A 7-year sample of DHS data (beginning in 2015) was obtained for each region of Sub-Saharan African countries in order to obtain a representative sample of recent data. Large sample sizes are used in these population-based, nationally representative surveys of every country.

### Population and eligibility criteria

Reproductive age women who are 15–49 years old and who had short birth interval in Sub-Saharan African countries were the source population. The study population was all the reproductive age women who were in the selected enumeration areas included in the analysis. Women who are not within the reproductive age range as defined by the study. Participants who did not have a short preceding birth interval, according to the study's specific definition of “short”, Survey responses that are incomplete or missing crucial data for the multilevel mixed-effects logistic regression analysis, data from women who did not consent to the use of their information for research purposes were excluded from this study.

### Data source and sampling procedure

To gain insight into pregnancy termination and determinates among women who had a short preceding pregnancy interval, 21 Sub-Saharan countries' DHS surveys were combined. Numerous datasets are used in each country's survey, including data on important health indicators like disease, mortality, use of family planning services, fertility, and access to maternity and child health care. Using a stratified two-stage cluster design, the Demographic and Health Survey first creates the enumeration areas and then generates a sample of households from each enumeration area in the second stage. The dependent and independent variables for each country were extracted using the individual record dataset (IR file) for this study and the data were subsequently appended using STATA. The outcome variable (termination of pregnancy) was generated by recoding the variables “ever had a terminated pregnancy” (v228) from the individual record (IR) data set. The study included a weighted sample of 283,785 women of reproductive age with a short interval between births (Table 1).

**Table 1:** Sample size for prevalence and determinants of termination of pregnancy among reproductive age women who had short preceding birth interval in Sub-Saharan Africa, DHS 2015–2022.

Country	Year of survey	Weighted sample (n)	Weighted sample (%)	World bank income classification, 2024
Angola	2015/2016	10,219	3.60	Lower-middle income
Benin	2017/2018	10,549	3.72	Low income
Burundi	2016/2017	12,288	4.33	Low income
Cameroon	2018	11,055	3.90	Lower-middle income
Ethiopia	2016	22,486	7.92	Low income
Gabon	2019	15,994	5.64	Upper-middle income
Gambia	2019/2020	16,160	5.69	Low income
Guinea	2018	13,984	4.93	Low income
Kenya	2022	41,576	14.65	Lower-middle income
Liberia	2019/2020	9,824	3.46	Low income
Mali	2018	7,264	2.56	Low income
Malawi	2015/2016	14,255	5.02	Low income
Nigeria	2018	29,630	10.44	Lower-middle income
Rwanda	2019/2020	9,952	3.51	Low income
Senegal	2019	5,999	2.11	Lower-middle income
Sera lion	2019	9,936	3.50	Low income
Tanzania	2022	9,651	3.40	Low income
Uganda	2016	13,064	4.60	Low income
South Africa	2016	5,382	1.90	Upper-middle income
Zambia	2018	8,622	3.04	Lower-middle income
Zimbabwe	2015	5,895	2.08	Low income
Total sample size		283,785	100	

### Study variables

#### Dependent variables

Pregnancy termination was the study's outcome variable, and it was generated from the individual record (IR) data set. The response option for the outcome variable “Have you ever had a terminated pregnancy” was binary: “Yes” meant the woman had had an abortion, and “No” meant she hadn't during the study period (32, 33).

#### Short birth interval

A short birth interval takes place when two consecutive live births occur less than 33 months apart. In following WHO guidelines, a non-short birth interval was defined as a previous birth interval greater than 33 months. By subtracting the first child's birthdate from the second child's birthdate, the birth interval was computed (34, 35).

#### Independent variables

**Figure 1:**
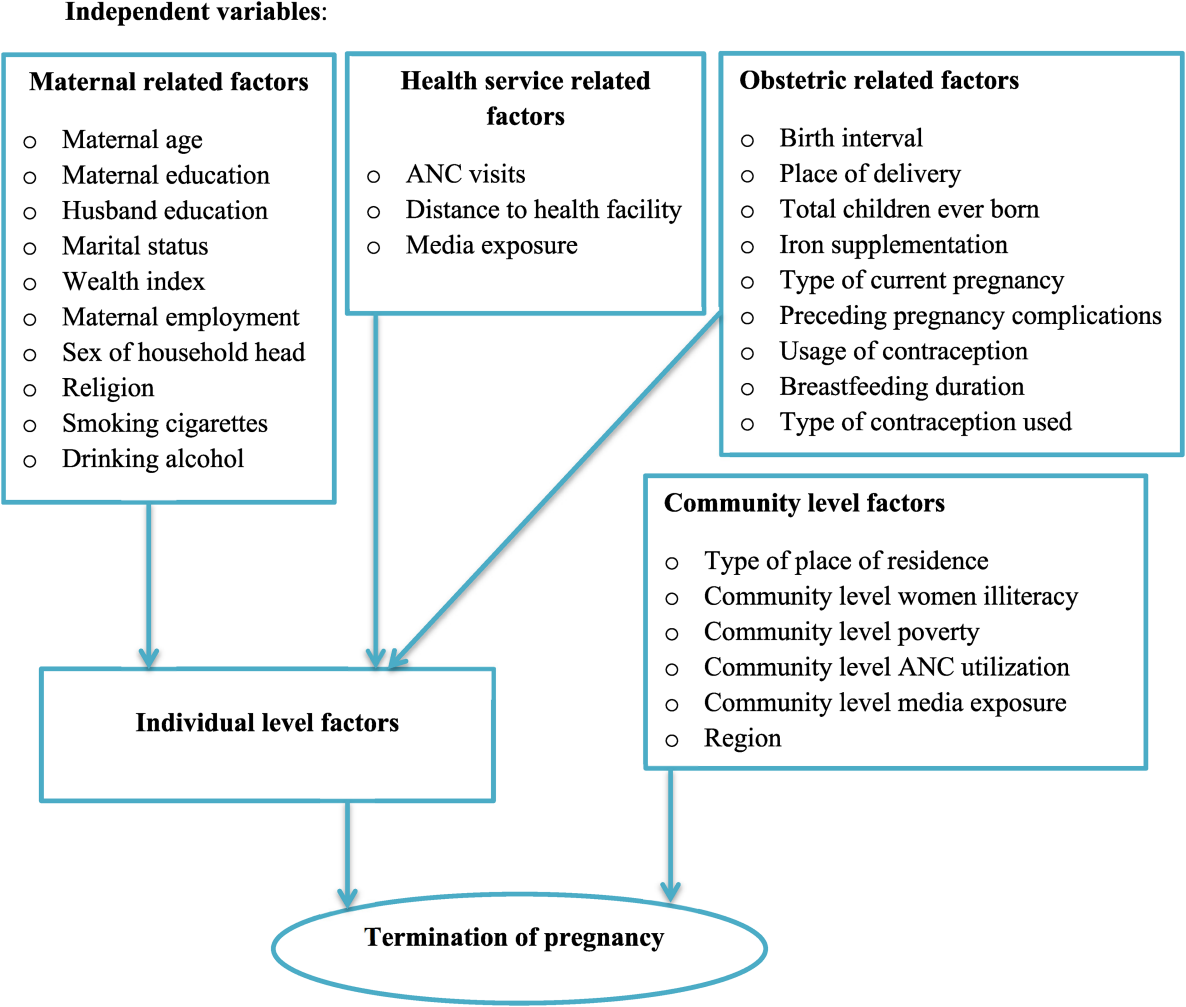
Conceptual framework for individual and community level factors associated with of termination of pregnancy among reproductive-age women who had short preceding birth interval in Sub-Saharan Africa.

Two sources of independent variables (individual and community-level variables) were included for this analysis because DHS data are hierarchical in nature. The individual-level independent variables were: sex of preceding birth (male, female) Sex of household head (male, female), Maternal age (15–24, 25–34, 35–49), Maternal educational status (no formal education, primary, secondary, higher), Husband educational status (no formal education, primary, secondary, higher), Religion (Orthodox, Catholic, Protestant, Muslim, Others), Maternal employment (no working, governmental employee, self-employee), marital status of the mother (unmarried, married, ever married), pregnancy complications (no, yes), number of ANC visits (no visit, 1–3, ≥4), total children ever born (≤3, 4–6, 7–9, >9), duration of breastfeeding (<6 months, ≥6 months), usage of contraception(yes, no), type of contraception used (not used, modern, traditional), household wealth index (poor, middle, rich), distance to health facility (big problem, not big problem), household media exposure (no, yes), smoking (no, yes), drinking alcohol (no, yes), previous mode of delivery (vaginal, caesarean section), type of current pregnancy (single, multiple). The community-level variables were place of residence (urban or rural), community-level women's illiteracy (low or high), community-level poverty (low or high), community-level media exposure (low or high), and community-level ANC utilization (low or high) (Figure 1).

### Data processing and statistical analysis

Statistical software (STATA/SE) version 14 was used to clean, record, and analyze the data once it had been extracted from recent DHS data sets. Prior to conducting any statistical analysis, the data were weighted using the sampling weight, primary sampling unit, and stratum in order to restore the survey's representativeness and account for the sampling design when computing standard errors to produce accurate statistical estimations. We used the weighting variable (v005) as a relative weight normalized to make the analysis survey-specific, while for the pooled data, we denormalized reproductive age women's individual standard weight variable by dividing the women's individual standard weight by the sampling fraction of each country: (women adjusted weight = V005× (total women aged 15–59 years in the country at the time of the survey)/(number of women aged 15–59 years in the survey).

The assumptions of standard logistic regression model such as independence observations and equal variance are broken due to the hierarchical nature of the DHS data. Mothers, for example, are nested within clusters, and we believe that those who participated in one cluster may have similar characteristics to those in another, which goes against the equal variance and independence assumptions between clusters in the ordinal logistic regression model. This implies that accounting for between-cluster effects requires the use of a complex model. In light of this, multilevel mixed-effects logistic regression was applied to identify the variables associated with pregnancy termination. Multilevel mixed effect logistic regression uses four models: the null model (outcome variable only), model I (only individual level variables), model II (only community level variables), and model III (both individual and community level variables).

The null model, which lacks independent variables, was employed to investigate the variation in abortion rates within the cluster. Evaluations were conducted on the relationships between the outcome variable (Model I) and the factors at the individual and community levels (Model II). The association between the community- and individual-level variables and the outcome variable was fitted simultaneously in the final model (Model III). To manage potential confounding, a multivariable analysis has been performed. To mitigate multicollinearity issues the Variance Inflation Factor (VIF) for all independent variables has been conducted and we have systematically removed those with VIF values exceeding 10. Additionally, we set a criterion for including variables in the final model at a *p*-value threshold of < 0.25. Applying a binary logistic regression model, the variables associated with termination of pregnancy were identified. Factors associated with pregnancy termination were expressed as an adjusted odds ratio (AOR) with a 95% significance threshold. In multivariable logistic regression, all variables with *p* values <0.05 were deemed statistically significant.

### Random effects

Random effects or measures of variation such as Likelihood Ratio test (LR), Intra-class Correlation Coefficient (ICC), and Median Odds Ratio (MOR) were computed to measure the variation of abortion across clusters. Taking clusters as a random variable, the ICC quantifies the degree of heterogeneity of termination of pregnancy between clusters (the proportion of the total observed variation in termination of pregnancy that is attributable to between cluster variations) (36) is computed as; $\mathrm{ICC}\;=\;\frac{\mathrm{VC}}{\mathrm{VC}+3.29}\times 100\text{\%}$. The Median Odds Ratio (MOR) is the median value of the odds ratio which quantifies the variation or heterogeneity in abortion between clusters in terms of odds ratio scale and is defined as the median value of the odds ratio between the cluster at high likelihood of abortion and cluster at lower risk when randomly picking out individuals from two clusters (37); MOR = *e* ^0.95√VC^.

Moreover, the PCV demonstrates the variation in the termination of pregnancy explained by determinants and computed as; $\;\mathrm{PCV}=\frac{\mathrm{Vnull}-\mathrm{Vc}}{\mathrm{Vnull}}\times 100\text{\%}$; where Vnull = variance of the null model and VC = cluster level variance (38). The fixed effects were used to estimate the association between the likelihood of abortion and individual and community level independent variables. It was assessed and the strength was presented using adjusted odds ratio (AOR) and 95% confidence intervals with a *p*-value of <0.05. Deviance = −2 (log likelihood ratio) was used to compare the models due to the nested nature of the model; the model with the lowest deviance and the highest log likelihood ratio has been selected as the best-fit model.

### Ethical approval and consent to participate

Since this study is merely a secondary review of the DHS data, ethical approval is not needed. We enrolled with the DHS web archive, requested the dataset for our study, and were granted permission to view and download the data files. As per the DHS study, all participant data were anonymized at the time of survey data collection. Visit in order to understand more about DHS data and ethical standards https://www.dhsprogram.com.

## Result

### Socio-demographic and economic characteristics of women who had short birth interval in Sub-Saharan Africa, DHS 2015–2022

A total of 283,785 women who had short birth intervals were included in this study. A quarter of women 70,122 (24.71%) had no formal education. More than half 6,909 (56.20%) of the women had pregnancy complications, and about 165,709 (58.39%) were living in rural areas of Sub-Saharan African countries. About 14,041 (4.95%) of women did not have ANC visits. More than half 145,376 (51.23%) of women living in Sub-Saharan African countries have high community poverty (Table 2).

**Table 2:** Socio-demographic, economic, and obstetric characteristics of women who had short preceding birth interval in Sub-Saharan Africa, DHS 2015–2022.

Variables	Frequency (*n*)	Percent (%)
Individual level variables		
Sex of preceding birth		
Male	82,261	51.01
Female	79,010	48.99
Maternal age		
15–19	89,876	31.67
20–35	139,015	48.99
36–49	54,894	19.34
Maternal educational level		
No formal education	70,122	24.71
Primary	83,885	29.56
Secondary	108,299	38.16
Higher	21,479	7.57
Husband educational level		
No formal education	50,263	35.46
Primary	37,197	26.24
Secondary	39,237	27.68
Higher	15,040	10.61
Maternal occupational status		
Not working	119,293	44.23
Governmental employee	70,641	26.19
Self-employee	79,782	29.58
Religion		
Orthodox	103,863	36.60
Catholic	65,556	23.10
Protestant	43,643	15.38
Muslim	22,230	7.83
Others	48,493	17.09
Marital status of the mother		
Never married	119,388	42.07
Currently married	143,670	50.63
Formerly/ever married	20,727	7.30
Smoking		
No	258,268	91.01
Yes	25,517	8.99
Drinking alcohol		
No	275,309	97.01
Yes	8,476	2.99
Previous mode of delivery		
Vaginal	103,564	93.74
Cesarean section	6,914	6.26
Pregnancy complications		
No	5,384	43.80
Yes	6,909	56.20
Sex of household head		
Male	196,128	69.11
Female	87,657	30.89
Distance to health facility		
Big problem	91,810	35.86
Not a big problem	164,185	64.14
Number of ANC visits		
No visit	14,041	4.95
1–3	56,910	20.05
≥4	212,834	75.00
Total children ever born		
≤3	220,149	77.58
4–6	38,927	13.72
7–9	17,103	6.03
>9	7,606	2.68
Duration of breastfeeding		
<6 months	54,718	19.28
≥6 months	229,067	80.72
Usage of contraception		
Yes	153,334	54.85
No	126,230	45.15
Type of contraception used		
No using	220,374	78.83
Modern	53,195	19.03
Traditional	5,995	2.14
Household wealth index		
Poor	112,539	39.66
Middle	52,853	18.62
Rich	118,393	41.72
Household media exposure		
No	84,321	29.71
Yes	199,464	70.29
Community level variables		
Place of residence		
Rural	165,709	58.39
Urban	118,076	41.61
Community media exposure		
Low	152,088	53.59
High	131,697	46.41
Community poverty		
Low	138,409	48.77
High	145,376	51.23
Community women's illiteracy		
Low	119,620	42.15
High	164,165	57.85
Community ANC utilization		
Low	120,274	42.38
High	163,511	57.62
Country category		
Central	37,268	13.13
Eastern	123,534	43.53
West	111,602	39.33
Southern	11,381	4.01

### Prevalence of termination of pregnancy among women who had short preceding birth interval in Sub-Saharan Africa countries

**Figure 2:**
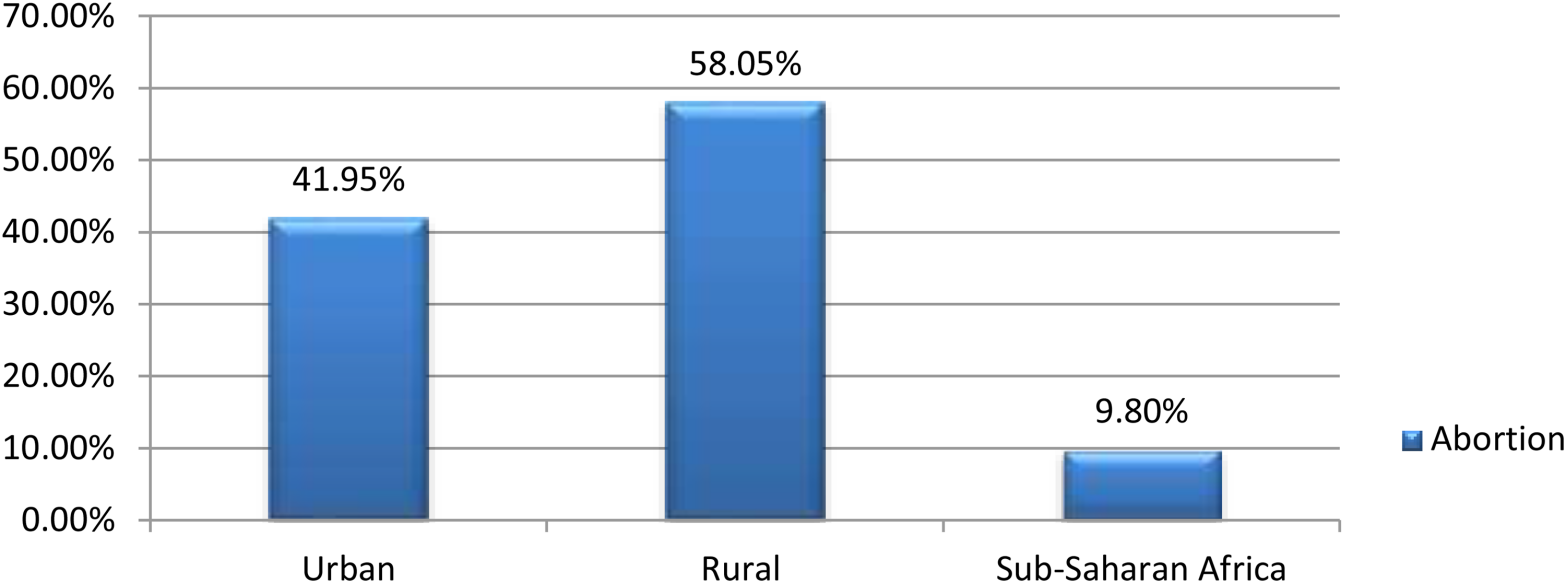
Prevalence of termination of pregnancy among reproductive age women who had short preceding birth interval in Sub-Saharan Africa.

**Figure 3:**
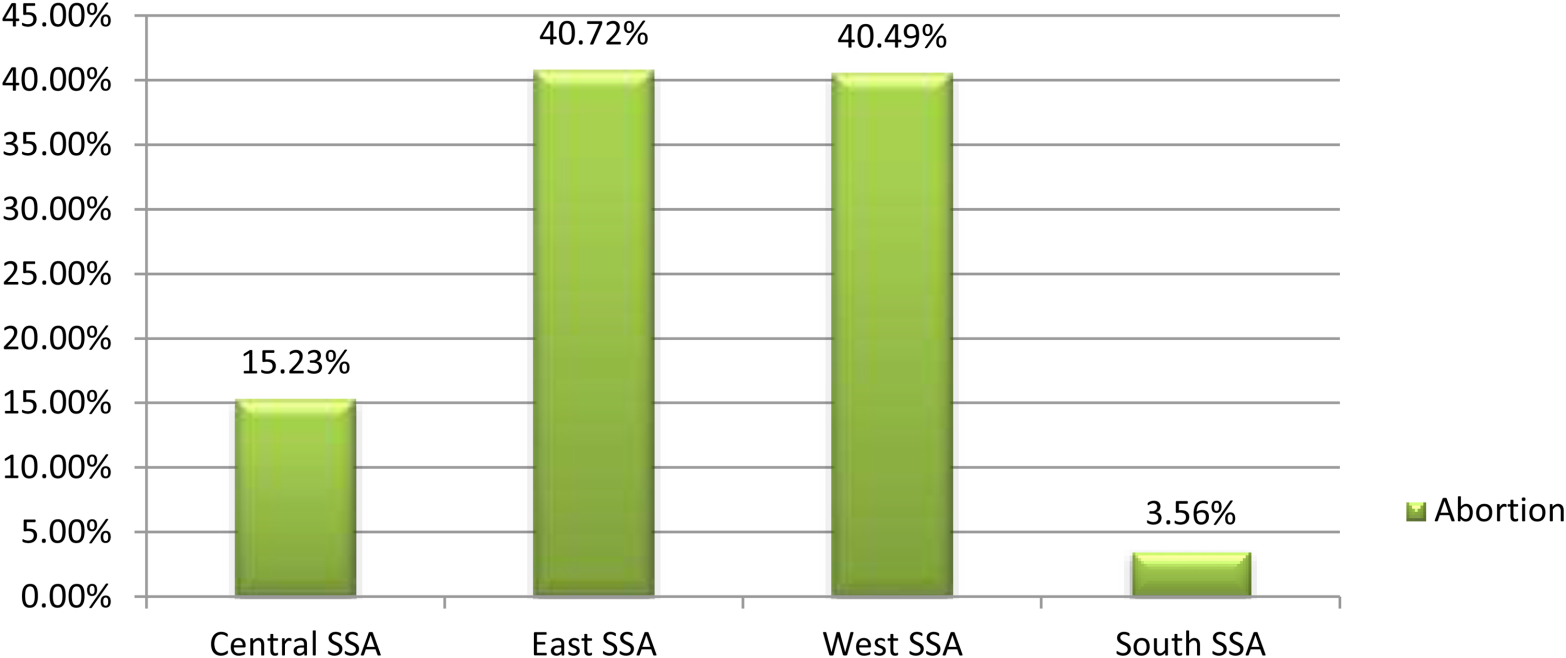
Regional prevalence of termination of pregnancy among women who had short preceding birth interval in Sub-Saharan Africa.

The prevalence of termination of pregnancy among women who had short birth interval in Sub-Saharan African countries was 9.80% [95% CI: (9.69, 9.91)]. The magnitude of urban and rural abortion in Sub-Saharan African countries was found to be 41.95% and 58.05%, respectively (Figure 2). East Sub-Saharan Africa (40.72%) and Southern Sub-Saharan Africa (3.560%) countries had the highest and lowest rates of termination of pregnancy, respectively (Figure 3).

### Measures of variation and model fitness

Findings from the null model showed that there were significant differences in termination of pregnancy between communities, with a variance of 0.0661827. In the null model, about 1.97% of the total variation on termination of pregnancy was occurred at the cluster level and is attributable to the community-level factors. In addition, the null model also had the highest median odds ratio (MOR) value (2.17) indicating when randomly select an individual from one cluster with a higher risk of termination of pregnancy and the other cluster at lower risk, individuals at the cluster with a higher risk of termination of pregnancy had 2.17 times higher odds of having termination of pregnancy as compared with their counterparts. The intraclass correlation value for Model I indicated that 1.81% of the variation in termination of pregnancy accounts for the disparities between communities. Next, we built Model II using community-level variables and the null model. Cluster variations were the basis for 1.79% of the differences in abortion, according to the ICC value from Model II. In the final model (model III), which attributed approximately 9.56% of the variation in the likelihood of termination of pregnancy to both individual and community-level factors. The model fitness was conducted by using log likelihood ratio and deviance in the final model (model III), which was the best-fitted model since it had the lowest deviance (6,054.7712) and the highest log likelihood ratio (−3,027.3856) (Table 3).

**Table 3:** Model comparison and random effect analysis for termination of pregnancy among women who had short preceding birth intervals in Sub-Saharan Africa, DHS 2015–2022.

Parameter	Null model	Model I	Model II	Model III
Variance	0.0661827	0.06079508	0.0600296	0.05985257
ICC	1.97%	1.81%	1.79%	1.78%
MOR	2.17	1.27	1.26	1.25
PCV	Reference	8.14%	9.30%	9.56%
Model fitness				
LLR	−89,440.022	−3,031.9665	−89,423.622	−3,027.3856
Deviance	178,880.022	6,063.933	178,847.244	6,054.7712

ICC, interacluster correlation; LLR, log likelihood ratio; MOR, median odds ratio; PCV, proportional change in variance.

### Association of individual and community-level determinants and abortion among reproductive age women in Sub-Saharan Africa

Sex of preceding birth, maternal age, presence of pregnancy complications, no ANC visits, previous mode of delivery, duration of breastfeeding, type of contraception used, poor household wealth status, and urban residence were significantly associated with termination of pregnancy among reproductive age women who had short birth intervals at a *p*-value of <0.05 in multivariable multilevel mixed-effect logistic regression analysis, where both the individual and community level factors were fitted simultaneously.

The odds of pregnancy termination were 1.21 times higher among women whose preceding birth was male compared to women whose preceding birth was female (AOR = 1.21, 95% CI: 1.05, 1.40). The odds of pregnancy termination were 1.57 times higher among women whose ages were 36–49 years compared to women whose ages were 15–19 years (AOR = 1.57, 95% CI: 1.27, 1.95). The odds of pregnancy termination were 1.28 times higher among women who had pregnancy complications as compared to women who did not have pregnancy complications (AOR = 1.28, 95% CI: 1.09, 1.49). Termination of pregnancy was 2.29 times higher among women who had no ANC visits as compared to women who had four or more ANC visits during pregnancy (AOR = 2.29, 95% CI: 1.26, 4.14).

The odds of pregnancy termination were 1.56 times higher among women who breastfeed as compared to women who did not feed (AOR = 1.56, 95% CI: 1.35, 1.81). The odds of pregnancy termination were 1.67 times higher among women who used traditional contraceptive methods as compared to women who modern contraceptive methods (AOR = 1.67, 95% CI: 1.13, 2.46). The odds of pregnancy termination were 1.74 times higher among women whose previous mode of delivery was cesarean section as compared to women who had vaginal delivery. (AOR = 1.74, 95% CI: 1.32, 2.30). Termination of pregnancy among reproductive age women who lives in poor wealth status was 1.50 times higher as compared to rich women (AOR = 1.50, 95% CI: 1.22, 1.85). The odds of pregnancy termination were 1.31 times higher among women whose place of residence was urban as compared to women from rural areas (AOR = 1.31, 95% CI: 1.06, 1.62) (Table 4).

**Table 4:** Multivariable multilevel logistic regression analysis of individual-level and community level determinants of pregnancy termination among women who had preceding short birth intervals in Sub-Saharan Africa, DHS 2015–2022.

Individual and community level variables	Model I AOR (95% CI)	Model II AOR (95% CI)	Model III AOR (95% CI)	*P*-value
Sex of preceding birth				
Male	1.22 (1.06, 1.40)		**1.21** **(1.05, 1.40)**	0.007
Female	1		1	
Maternal age				
15–19	0.56 (0.40, 1.79)		0.57 (0.40, 1.80)	0.081
20–35	1		1	
36–49	1.59 (1.29, 1.96)		**1.57** (**1.27, 1.95)**	0.000
Sex of household head				
Male	1		1	
Female	1.01 (0.85, 1.20)		1.00 (0.84, 1.20)	0.916
Maternal educational level				
No formal education	0.94 (0.63, 1.40)		0.98 (0.66, 1.46)	0.671
Primary	0.95 (0.65, 1.39)		0.99 (0.67, 1.45)	0.735
Secondary	1.10 (0.77,1.58)		1.11 (0.77, 1.59)	0.796
Above	1		1	
Husband educational level				
No formal education	1.39 (0.99, 1.92)		1.40 (0.01, 1.94)	0.074
Primary	1.35 (0.97, 1.87)		1.37 (0.98, 1.91)	0.062
Secondary	1.39 (0.03, 1.89)		1.37 (0.01, 1.87)	0.99
Above	1		1	
Maternal occupation				
Not working	0.76 (0.64, 1.90)		0.75 (0.64, 1.89)	0.071
Governmental employee	1		1	
Self-employee	0.84 (0.67, 1.06)		0.84 (0.67, 1.05)	0.112
Religion				
Orthodox	1		1	
Catholic	1.27 (0.86, 1.88)		1.25 (0.84, 1.85)	0.258
Protestant	0.92 (0.74, 1.14)		0.90 (0.72, 1.13)	0.350
Muslim	0.78 (0.62, 0.98)		0.79 (0.63, 1.00)	0.051
Others	0.91 (0.66, 1.28)		0.86 (0.61, 1.20)	0.314
Pregnancy complications				
No	1		1	
Yes	1.28 (1.10, 1.50)		**1.28** (**1.09, 1.49)**	0.001
Distance to health facility				
Big problem	0.97 (0.83, 1.13)		0.98 (0.84, 1.15)	0.873
Not a big problem	1		1	
Number of ANC visits				
No visit	2.29 (1.27, 4.14)		**2.29** (**1.26, 4.14)**	0.009
1–3	0.99 (0.83, 1.17)		1.01 (0.851.20)	0.944
≥4	1		1	
Duration of breastfeeding				
<6 months	1.57 (1.36, 1.82)		**1.56** (**1.35, 1.81)**	0.000
≥6 months	1		1	
Usage of contraception				
Yes	0.98 (0.82, 1.17)		0.99 (0.83, 1.18)	0.948
No	1		1	
Type of contraception used				
Not used	1.15 (0.94, 1.39)		1.15 (0.94, 1.39)	0.221
Modern	1		1	
Traditional	1.67 (1.14, 2.47)		**1.67** (**1.13, 2.46)**	0.010
Previous mode of delivery				
Vaginal	1		1	
Cesarean section	1.75 (1.33, 2.31)		**1.74** (**1.32, 2.30)**	0.000
Total children ever born				
1–3	1		1	
4–6	1.31 (0.09, 1.57)		1.30 (0.08, 1.56)	0.075
7–9	1.34 (0.02, 1.76)		1.36 (0.03, 1.78)	0.097
>9	0.81 (0.50, 1.33)		0.82 (0.50, 1.34)	0.397
Household wealth index				
Poor	1.39 (1.14, 1.71)		**1.50** (**1.22, 1.85)**	0.001
Middle	1.32 (1.04, 1.67)		1.39 (0.09, 1.76)	0.095
Rich	1		1	
Household media exposure				
No	0.74 (0.62, 0.89)		0.74 (0.61, 1.89)	0.065
Yes	1		1	
Community level variables				
Place of residence				
Rural		1	1	
Urban		1.03 (0.99, 1.05)	**1.31** (**1.06, 1.62)**	0.035
Community level media exposure				
Low		1.14 (1.09, 1.20)	1.20 (0.93, 1.54)	0.434
High		1	1	
Community poverty				
Low		1	1	
High		0.96 (0.91, 1.01)	0.90 (0.69, 1.14)	0.724
Community women's illiteracy				
Low		1	1	
High		0.96 (0.91, 1.01)	1.05 (0.83, 1.34)	0.182
Community level ANC utilization				
Low		0.96 (0.92, 1.00)	1.03(0.82, 1.29)	0.857
High		1	1	

NB, variables such as marital status of the mother, smoking, drinking alcohol, and country category were not included in the final fitted model analysis due to a violation of the chi-square assumption and the presence of multicollinearity.

Bold values indicate significantly associated variables with the outcome variable.

## Discussion

This study aimed to assess the prevalence and determinants of pregnancy termination among reproductive-age women who had short birth intervals in Sub-Saharan Africa using 2015–2022 Demography and Health Survey data from each country. In this study, the prevalence of pregnancy termination among women who had short birth interval in Sub-Saharan African countries was 9.80% (95% CI: 9.69, 9.91). This finding was consistent with a previous study conducted in Ethiopia, 9.8% (33), Sierra Leone, 9% (39), and Ghana, 9% (40). The prevalence of pregnancy termination in this study was higher than the findings conducted in Nigeria, 4.9% (41), East Africa, 5.96% (32), Brazil, 4.5% (42), and Iran, 3.8% (43). These discrepancies could be explained by variations in health policies, healthcare quality, and socioeconomic and cultural differences between countries. The higher percentage of pregnancy termination in our analysis indicates that this issue needs to be given more attention in Sub-Saharan Africa.

On the other hand, the prevalence of pregnancy termination in this study was lower than the findings conducted in Ethiopia, 14.5% (44), Cameroon, 21% (45), Egypt, 21% (46), Nepal, 21.1% (20), and Mozambique, 25% (40). A plausible reason might be attributed to variations in the study period and study population, as well as the progressive enhancement of maternal health care service accessibility and utilization.

In multivariable, multilevel mixed-effect logistic regression analysis, sex of preceding birth, maternal age, presence of pregnancy complications, no ANC visits, previous caesarean section delivery, duration of breastfeeding, type of contraception used, poor household wealth status, and urban residence were found to be significantly associated with pregnancy termination among reproductive-age women who had short birth intervals.

Compared to women whose preceding birth was female, the odds of terminating a pregnancy were 1.21 times greater for women whose previous birth was male. This finding is consistent with the studies conducted in Sub-Saharan Africa (47, 48). The explanation could be cultural Gender preferences, particularly the desire for a male child, can significantly influence pregnancy termination decisions, especially in societies where sons are valued for economic, social, or cultural reasons. Families may feel compelled to continue pregnancies until a male child is born, resulting in higher rates of pregnancy termination, particularly when previous children are female or if there is a perceived need for gender balance. This practice can lead to gender imbalances within populations, affecting social structures and perpetuating discriminatory attitudes toward women. Moreover, the societal pressure to conform to these gender norms can restrict women's reproductive autonomy, reinforcing patriarchal values that undervalue female children and contribute to a cycle of gender-based discrimination that persists across generations. This dynamic not only has implications for individual families but also poses significant challenges to gender equality, with long-term effects on women's health, empowerment, and societal roles (48). The other explanation could be family balancing: Some families may seek a balance in the genders of their children. If a family already has one or more male children, they may choose to terminate a subsequent pregnancy if prenatal testing indicates another male child, in the hope of having a female child in the future (49).

The odds of pregnancy termination were 1.76 times higher among women whose ages were 36–49 years compared to women whose ages were 15–19 years. It is in line with study findings in Ethiopia (50), Ghana (51, 52), Mozambique (40), Sierra Leone (39), and China (53). This may be due to the fact that older women have an increased risk of cardiovascular disease, diabetes mellitus, and chromosomal abnormalities, making them more likely to experience pregnancy complications that could worsen pregnancy terminations (54). On the contrary, studies from Arba Minch and Wolayita Sodo town, Ethiopia (55), East Africa (56), and Chile (57) revealed a strong association between pregnancy termination and younger women age. The explanation might be that adolescent girls and young women are ignorant of safe sex practices, such as how to avoid unwanted pregnancy and complications related to pregnancy termination. Another explanation is that younger women don't know as much about family planning as older women do.

The odds of pregnancy termination were 1.28 times higher among women who had pregnancy complications as compared to women who did not have pregnancy complications. This finding is consistent with previous findings in Iran (58), the United Kingdom (59), Pennsylvania (60), and Canada (61). The possible explanation be due to most pregnancy complications are associated with decreased placental nutrient, which results placental insufficiency and these complications are associated with pregnancy termination, preterm birth and perinatal death (62).

Pregnancy termination was 2.40 times higher among women who had no ANC visits as compared to women who had four or more ANC visits during pregnancy. It is in line with the previous studies conducted in Ethiopia (63, 64), Uganda (65, 66), and Nigeria (67, 68). The possible explanation could be that the lack of ANC visits can hinder the timely detection and management of pregnancy-related issues. During these visits, healthcare providers can identify complications early and offer appropriate interventions, which are crucial in reducing the risk of pregnancy termination. The importance of optimal ANC visits cannot be overstated, as they significantly enhance the likelihood of receiving high-quality care throughout pregnancy, thereby preventing spontaneous pregnancy terminations. Furthermore, rural women often struggle with geographic distances, transportation issues, and socio-cultural factors that hinder their access. In urban areas, challenges such as overcrowded facilities, long waiting times, and economic constraints can also deter timely care (69, 70). Therefore, public health strategies must focus on enhancing ANC services, especially for women in disadvantaged communities.

The odds of pregnancy termination were 1.56 times higher among women who breastfeed for less than 6 months as compared to women who breastfeed for 6 and more months. This finding is in line with the studies conducted in low and middle income countries (49), Canada (71), and The USA (72). The association between short breastfeeding periods and pregnancy termination can be complex and multifaceted. Some of the potential justifications and factors that might explain this association could be breastfeeding as a natural contraceptive: Breastfeeding, especially exclusive breastfeeding, can act as a natural contraceptive method known as lactational amenorrhea, which can delay the return of fertility and menstruation. However, this method is not completely reliable, and its effectiveness can decrease with time, particularly after 6 months or when supplemental feeding is introduced (49). Another justification could be socioeconomic and health factors: Women who breastfeed for shorter periods may do so due to socioeconomic reasons, such as the need to return to work, or health issues that prevent prolonged breastfeeding. These same factors could also contribute to the decision or need for pregnancy termination (73).

Compared to women who utilized modern contraceptive methods, women who used traditional contraceptive methods had 1.67 times higher odds of terminating their pregnancy. This is corroborated by previous studies carried out in (74–76). The association between the use of traditional contraceptive methods and a higher likelihood of pregnancy termination, as compared to the use of modern contraceptive methods, can be attributed to several factors. This justification could be contraceptive effectiveness: Traditional contraceptive methods are generally less effective than modern methods. This lower effectiveness can lead to a higher incidence of unintended pregnancies, which may result in a greater number of pregnancy terminations (47). The other explanation might be personal preferences: Some women may choose traditional methods due to personal or religious beliefs, or due to concerns about the side effects of modern contraceptives. However, this choice may increase the risk of unintended pregnancies and the potential for pregnancy termination (77).

Furthermore, traditional methods often have lower effectiveness, leading to a higher incidence of unintended pregnancies and, consequently, increased pregnancy termination rates. Additionally, personal and religious beliefs significantly influence contraceptive choices; women may prefer traditional methods due to perceptions of safety or cultural acceptability, despite these methods being associated with higher risks of unintended pregnancies (41, 78).

The acceptability of contraception among women is a pivotal factor in the context of pregnancy termination. When contraception is widely accepted and utilized, the rate of unintended pregnancies tends to decrease, subsequently leading to a reduction in the number of pregnancy terminations. Conversely, when women are reluctant to use contraception due to personal beliefs, societal pressures, or lack of access the incidence of unintended pregnancies can increase, potentially resulting in a higher rate of pregnancy terminations (47). This association is further complicated by the influence of partners or spouses, whose acceptance or refusal of contraception can significantly impact a woman's reproductive choices. Therefore, understanding the nuances of contraception acceptability is crucial for public health initiatives aimed at reducing the prevalence of pregnancy termination by addressing the root causes of unintended pregnancies (79–81).

The odds of pregnancy termination were 1.71 times higher among women whose previous mode of delivery was caesarean section as compared to women who had vaginal delivery. It is consistent with studies in France (82) and Scotland (83), however to date, this finding is conflicting the study conducted in Germany (84). Although the exact causes of the link between pregnancy termination and Caesarean delivery are unknown, placental abnormalities may be implicated. But frequently, there is no clear underlying reason for these unfavorable incidences (85). According to this study's analysis of wealth status, women from the poorest homes were 1.50 times more likely than those from the richest households to undergo pregnancy termination. This is consistent with the studies conducted in Nepal (20), India (86), and China (87). The possible explanation could be that women from low-income households have inadequate access to healthcare, poor health care practices, and poor health-seeking behaviour. As a result, the women have a higher probability of pregnancy termination.

The odds of pregnancy termination were 1.31 times higher among women whose place of residence was urban as compared to women from rural areas. This is supported by the previous studies conducted in Ethiopia (19, 55, 88, 89), Cambodia (90), Ghana (91), and India (92). The possible explanation might be that, compared to women in rural areas, women in urban areas have more access to abortion services. In the same manner, premarital sex is more common in urban settings and can result in induced abortions and unwanted pregnancies. The finding of this study is inconsistent with the studies conducted in China (87), and Vietnam (93) where pregnancy termination is strongly associated with women from rural areas. This could be because rural areas lack access to media, which could lead to restricted information regarding abortion complications. It could also be because women are less likely to use maternal health care services, such as family planning, ANC visits, understanding of pregnancy signs, and birth planning.

The study's strength was the utilization of recently conducted large-sample national demography and health surveys from 21 Sub-Saharan African countries. Another strength of this study was the use of mixed multilevel logistic regression to determine two-level factors (individual and community-level factors), which could not be done using ordinary logistic regression. However, the study is limited by its reliance on secondary data, which may lack key variables, including maternal psychological factors that could influence the outcome variables. Additionally, potential biases, such as reporting bias in the DHS data, may further affect our findings.

## Conclusions and recommendation

This study concludes that pregnancy termination rates among women with short birth intervals are high. The study identified that both individual and community-level variables were determinants of pregnancy termination. To effectively reduce pregnancy termination rates in Sub-Saharan Africa, ministries of health should implement targeted interventions that focus on enhancing antenatal care service utilization among women, particularly those from urban areas. This could involve developing comprehensive outreach programs that promote awareness of available services, providing incentives for women to attend antenatal visits, and establishing collaborations with local community organizations to ensure that tailored support reaches those most in need. We also suggested that future research could incorporate qualitative studies to further explore gender preferences and their influence on pregnancy termination.

## Data Availability

The raw data supporting the conclusions of this article will be made available by the authors, without undue reservation.
